# Facilitators and Barriers Surrounding the Role of Administration in Employee Job Satisfaction in Long-Term Care Facilities: A Systematic Review

**DOI:** 10.3390/healthcare8040360

**Published:** 2020-09-24

**Authors:** Kimberly Lee, Michael Mileski, Joanna Fohn, Leah Frye, Lisa Brooks

**Affiliations:** School of Health Administration, Texas State University, 601 University Drive, San Marcos, TX 78666, USA; mileski@txstate.edu (M.M.); jtf64@txstate.edu (J.F.); lmf106@txstate.edu (L.F.); llb127@txstate.edu (L.B.)

**Keywords:** job satisfaction, associate engagement, long-term care, older adults, nursing homes, assisted living

## Abstract

Previous literature has shown how associate engagement has positively impacted on productivity, job satisfaction, safety, retention, consumer sentiment, and financial performance in hospitals and healthcare systems. However, a lack of research showing the relationship between associate engagement and job satisfaction within the long-term care environment has existed. Our objective was to investigate characteristics within the long-term care environment that promote and detract from associate job satisfaction and extrapolate the best practices in maintaining job satisfaction and engagement. This systematic review queried CINAHL, PubMed (MEDLINE), and Academic Search Ultimate databases for peer-reviewed publications for facilitators and barriers commensurate with employee job satisfaction in long-term care facilities using the Preferred Reporting Items for Systematic Reviews and Meta-Analysis (PRISMA) and the Kruse Protocols. The authors identified 11 facilitators for job satisfaction and 18 barriers to job satisfaction in the 60 selected articles. The top four facilitators were Supportive Leadership, Capable and Motivated Employees, Positive Organizational Values, and Social Support Mechanisms. The top four barriers were condescending management style, high job demands, lack of self-care, and lack of training with medically complex patients. The systematic review revealed the importance of maintaining satisfied employees in the long-term care workplace through am emphasis leadership and on the facilitators identified to best serve their associates and improve care for residents.

## 1. Introduction and Background

### 1.1. Introduction

#### 1.1.1. Rapid Growth of 65+ Population

The fastest growing segment of the national and world population is the 65 years and over age bracket. World Population Ageing 2019 [1] emphasizes that the current world population of individuals at 65 years or greater numbers 703 million, with a projection of 1.5 billion in 2050. In the United States, the population of age 65 and over is at 52 million, with a projection of a doubled population of 95 million by 2060 [2,3]. In addition, a greater percentage of our total population in the US and in the world is comprised of people aged 65 and older [1,2,3], with a projected increase of 1 in 5 persons in the US and 1 in 6 persons in the world by 2050 [1,4]. In the United States, the number of those aged 65 and over will rise from the current 16% of total population to 23% of the total population by 2060 [2,3].

Technological and medical advances have further contributed to the expanding population of the 65-and-over age bracket, having led to the expectation that a senior citizen in developed country will live more than a quarter of their adult life after the age of 65. These trends apply to the United States and are included in all the world’s developed countries [1].

#### 1.1.2. Residential and Care Needs

With the growth in the 65-and-over population, one quickly observes the impact on the organizations providing residential and healthcare services. The National Center of Health Statistics has estimated that approximately 65,000 regulated long-term care service providers (including nursing homes, assisted living or similar residential communities, home health agencies, adult day care services, and hospice organizations) have cared for over 8.3 milling individuals in the United States [5]. Of the 65,000 service providers, 15,600 nursing homes and 28,900 assisted living or similar residential care facilities delivered care to 2.16 million residents. The estimated 1.5 million number of individuals aged 65 and over living in nursing or skilled nursing facilities is projected to rise to 2.3 million by 2030 and 3 million by 2060 [2,5]. The over-65 population residing in nursing homes and residential care communities come with a number of chronic diseases, with Alzheimer’s’ Disease, dementias, depression, and hypertension being most prevalent [5]. To care for an aging population with chronic medical needs, approximately 1.5 million, full-time nursing associates provide services to the residents of nursing homes and residential communities, requiring 3.80 h per resident in nursing homes and 2.64 h per resident in residential care communities [5].

#### 1.1.3. Burden on Society and Economics

A growing number of elders requiring care will result in a burden on both society in general and on the support systems that sustain their health and care. Seniors will outnumber children by 2035 [2]. The Population Reference Bureau (PRB) projects that by 2030, less than 3 working age adults in the US will support every US individual over 65, a decrease from over 4 US working adults to individuals of 65 and over in 2014 [2].

With the growth of the population of the 65-and-older bracket in the coming years, determining the best systems and practices to sustain a healthy, well, and productive aging population, while also providing quality care within residential care communities and nursing homes, has become paramount for this quickly expanding population. The United Nations (2019) emphasized the importance of prolonging the wellness of the gaining population by establishing the Sustainable Development Goals (SDGs) as outlined for sustainability of care, independence, and healthy aging of the growing demographic of the 65-and-over population by the United Nations Committee on Health [1]. Many current sources recognize the importance of established policies, technology, living environments, social support mechanisms, planning, and education to help the 65-and-over population to maintain independence and self-sufficiency for longer and to relieve the economic and social burden on the younger cohorts of society [1,2,3].

### 1.2. Background

#### 1.2.1. Job Satisfaction, Associate Engagement, and Organizational Outcomes

Previous research literature from various industries, including healthcare, has established the relationship between job satisfaction and associate engagement [6,7,8,9,10,11]. Most importantly, previous research literature focusing on the healthcare industry has also consistently established the relationship between associate engagement with organizational performance including customer and patient satisfaction in healthcare [7,8,9,10,12,13,14]. Most recently, in a special report on healthcare workforce engagement, Press Ganey [10] reported how associate engagement positively impacts on productivity, job satisfaction, safety, retention, consumer sentiment, and financial performance. The bulk of healthcare literature has been conducted with hospitals and health care systems being the primary source of the data [7,8,9,10,12,13,14,15].

Key facilitators of job satisfaction and engagement in hospitals and healthcare systems have included manager support and style [7,8,12,13,14], supportive workplace processes and technology [7,13,15], presence of a positive organizational culture based upon the mission and values of an organization [7,8,9,10,14], employee’s feelings of making a difference, including use of and development of skills [7,11,12,13,14], meaningful and positive workplace relationships and teaming [8,9,10,11,13,14], transformational leadership style [7,10,14], and frequent and transparent communication by leaders [7,8,9,10,14]. Supportive leadership included managers or leaders who both earned and extended trust between team members, demonstrated respect and caring [7,13,14], and promoted career development [11,14]. One source [12] cited supportive leadership as accounting for 63% influence over job satisfaction, compared to other factors. A positive organizational culture was defined to be mission- and values-based [7,8], promoting resilience and adaptation to change [10], inclusive of employee empowerment and shared governance [14], and based on safety, excellence, and quality of patient care [9,10,11,12,13,14,15].

Key barriers to job satisfaction and engagement in hospitals and health systems have included workload demands [14], limited resources and policies [11,14], limited salary and benefits [11], and more recent literature has included barriers such as presence of siloes, lack of consensus on goals, a lack of awareness on how systems integrate across functions and departments, a lack of frequent communication, and a lack of involvement around safety, quality, and the patient experience [9,10].

#### 1.2.2. Gap of Research in the Long-Term Care Environment

A lack of research and literature studying job satisfaction and associate engagement exists in the long-term care environment. Only recently have investigators or publications addressed long-term care specifically and acknowledged that the study of job satisfaction and/or associate engagement are critical in this setting. For example, the Centers of Medicare and Medicaid Services only recently allocated funds from the Civil Money Penalty Reinvestment Program to create a generic employee satisfaction survey, accompanying toolkits, and education materials on data collection and process-improvement strategies [16]. Thus, a gap exists in the research relative to the relationship between job satisfaction, associate engagement, the perception of quality care by residents in long-term care, and family satisfaction.

#### 1.2.3. Cost of Associate Disengagement

To further draw attention to the critical impact of job satisfaction and associate engagement, it is important to consider the impact of the disengaged and dissatisfied healthcare worker on Americans’ pocketbooks. One source reported that 68% of employees from all industries are not engaged and that this disengagement costs US employers $400 billion annually [15].

#### 1.2.4. Societal and Public Health Crises—Multiplier of Importance of Healthcare Associate Satisfaction

Crises such as public health pandemics (e.g., Covid-19) and natural disasters further exacerbate the sense of urgency in addressing morale and the satisfaction of healthcare team members in healthcare organizations. Recently, initial research findings and literature have emerged acknowledging the stress imposed by Covid-19 conditions on the healthcare workforce, impacting job satisfaction, morale, and risking the imposition of unintended negative impacts on patients and quality [17,18]. With the nursing home and other residential communities as epicenters of the Covid-19 pandemic, stress experienced by long-term care workers will certainly impact on patient satisfaction and quality of care.

### 1.3. Significance and Purpose

Given the quickly expanding over-65 population worldwide, an anticipated increased demand for nursing homes and residential care communities to care for the aging population, an increased burden on social and economic systems, an anticipated increase in need for nursing and care professionals, the relationship between nursing and healthcare professional’s job satisfaction and engagement with patient and organizational outcomes, and the impact of public health crises and pandemics (e.g., Covid-19), a sense of urgency exists to identify systems and practices that either facilitate or impede administrators in long-term care facilities to enhance job satisfaction and improve outcomes in quality of care and organizational effectiveness [1,2,3,4,5,6,7,8,9,10,11,12,13,14,15,16,17,18]. Further to this point, the investigators’ two key purposes in this study were to investigate characteristics within the long-term care environment that promote and detract from associate job satisfaction and extrapolate the best practices in maintaining job satisfaction and engagement, ultimately leading to best practices in creating a positive organizational culture for associates and a sound foundation for delivering resident-centered care within long-term care settings. The investigators used a systematic literature review method.

For the purposes of this study, the terms “associates”, “employees”, and “healthcare workers” were used synonymously. The investigators intentionally have utilized the word “associates” to be more inclusive, contemporary, and substantive in impacting culture. In addition, much has been written relating to job satisfaction with associate engagement. It is generally accepted that job satisfaction is a component and a contributing factor of overall associate engagement in the workplace. A number of published articles have substantiated this relationship [6,10,12,13,14].

## 2. Materials and Methods

**Figure 1:**
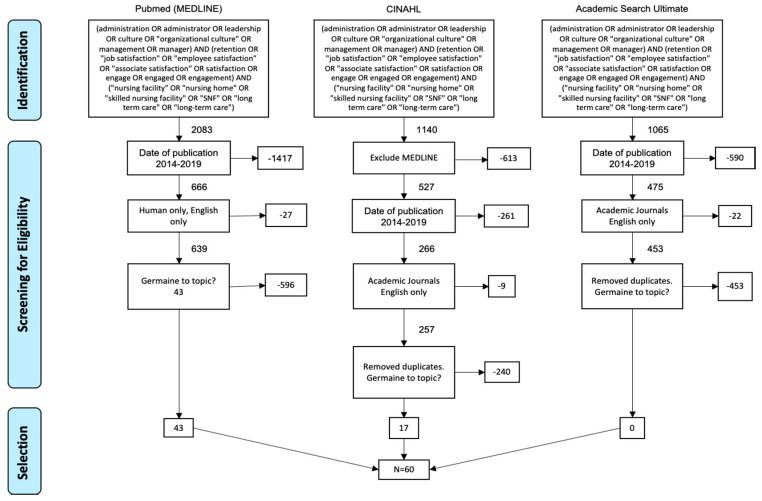
Preferred Reporting Items for Systematic Reviews and Meta-Analysis (PRISMA) Flow Chart.

This study was conducted utilizing the Preferred Reporting Items for Systematic Reviews and Meta-Analyses (PRISMA) guidelines and the Kruse Protocol for writing systematic reviews [19,20]. The initial search was conducted using Pubmed (MEDLINE), Cumulative Index of Nursing and Allied Health Literature (CINAHL), and Academic Search Ultimate. These databases were chosen due to their widespread use and availability. A Boolean search was conducted using a complex three-string search as included in Figure 1.

The summary of the selected articles included the Population, Intervention, Comparison, and Outcome (PICO) requirements for Prisma guidelines [19]. The reviewers read and analyzed the complete articles and noted the facilitators and barriers indicated to influence job satisfaction in long-term care facilities. Each article was completely assessed by at least two reviewers and the reviewers met routinely to arrive at a consensus on the facilitators, barriers, and themes of each article. Weekly consensus meetings occurred throughout the systematic review analysis. The reviewers/authors sought to reduce the risk of bias in the analysis technique through having multiple reviews and weekly consensus meetings.

### 2.1. Inclusion Criteria

Authors reviewed articles yielded from the search, determined germane literature, and summarized themes based on consensus. Inclusion criteria included English language and peer-reviewed articles. Articles must have been published in academic journals to be included. The dates of inclusion were 1 January 2014 through to 30 September 2019. To be included, articles must have explored the effects of administration/organizational culture on job satisfaction, employee satisfaction, or employee engagement in long-term care facilities.

### 2.2. Exclusion Criteria

Articles were only included in the study if they were determined to be germane by all authors. Trade industry reports were excluded. Poster presentations were excluded. Any works without a clear peer review process were excluded. Articles which were not specific to long-term care environments were excluded. Bias was not considered when reviewing research for this study. The final sample of articles after meeting exclusion criteria was analyzed and yielded a kappa statistic (k = 1), which showed perfect interrater agreement [21].

### 2.3. Study Selection

The initial search resulted in 4288 results. All duplicates were removed, leaving 3675 articles. Publication time frame was limited to between 2014 and 2019, leaving 1407 articles. Additionally, only academic journals and articles in the English language were included, leaving 1349 articles. Duplicates and non-germane articles were then removed from the results, leaving 60 articles for use in this qualitative analysis and review. Authors retained articles that only occurred within long-term care facilities and included facilitators or barriers to job satisfaction of associates or employees. A summary of the articles chosen for inclusion is included in Appendix A Table A1.

### 2.4. Data Analysis

Narrative summaries related to factors that influenced job/employee satisfaction, retention, and engagement regarding administration or organizational culture in long-term care environments were extracted from each article. Summaries were then grouped into larger recurring themes that identified either key determinants or impediments to job/employee satisfaction, retention, and engagement. The themes were chosen by consensus of the authors and were chosen as they provided overarching summaries to the facilitators and barriers extracted from the articles. Themes were then summarized via two affinity matrix tables, one for facilitators and one for barriers. Table 1 and Table 2 document the themes, their citation occurrence, their frequency sum, and frequency percentages.

## 3. Results

The 60 articles included in the qualitative analysis are summarized in Appendix A Table A1, with the most recent publications listed first by their primary authors. The 60 articles yielded 162 instances of facilitators and 97 instances of barriers relative to job satisfaction in long-term care facilities.

After a number of routine, weekly consensus meetings, the reviewers counted the number of times that a facilitator theme occurred in each article and consolidated the frequency and article references in an affinity matrix for further analysis in Table 1. Twelve facilitator themes emerged from the qualitative review of full articles, with a total of 162 occurrences. The top three facilitator themes accounted for 59.26% of the total occurrences. A theme of supportive leadership occurred 51 times (31.48%) [22,23,24,25,26,27,28,29,30,31,32,33,34,35,36,37,38,39,40,41,42,43,44,45,46,47,48]. Capable and motivated employees occurred 25 times (15.43%) [22,27,30,32,35,45,49,50,51,52,53,54,55,56,57,58,59,60]. Positive organizational values occurred 20 times (12.35%) [22,23,24,29,31,32,33,48,51,53,54,57,61,62,63,64] and social support mechanisms occurred 15 times (9.62%) [22,23,25,29,34,41,45,47,65,66,67,68]. Adequate job resources occurred 11 times (6.79%) [32,38,45,47,54,55,69,70,71] and career/professional development occurred 10 times (6.17%) [25,35,37,38,43,48,57,72,73]. Initial orientation and training [22,38,58,64,68,74] and patient-centered philosophy [22,28,48,73,75] both occurred 9 times (5.56%) respectively. Enjoyment of relationships with patients occurred 7 times (4.32%) [25,39,41,48,72,76], non-profit ownership occurred 4 times (2.47%) [53,55,63], and organizational systems and processes occurred once (0.62%) [22].

The authors/reviewers identified themes of barriers through qualitative analysis and consensus meetings. The authors/reviewers counted the number of times that a barrier theme occurred in each article and consolidated the frequency of occurrence in the 60 articles in an affinity matrix, illustrated in Table 2. Eighteen barrier themes were identified in the qualitative review and barriers occurred 98 times in the review of the 60 articles. Six of the eighteen barriers accounted for 61.22% of the total barriers. Condescending management style occurred 15 times (15.31%) [27,34,35,38,41,42,46,51,66,71,77]. High job demands, which included factors such as physical and psychological burdens [25], understaffing [38,46,67], heavy workloads [38,78], lack of time to complete tasks [40,43], limited staffing resources [46], reduced teamwork [46], unfair hours [67,71], regulations on nurse roll flexibility [72], and physical exhaustion [65], occurred 13 times (13.27%) [25,38,40,43,46,65,67,71,72,77,78]. Lack of self-care as exhibited by the associates occurred 9 times (9.18%) 30,33,33,46,47,54,59]. Lack of training with medically complex patients (8.16%) [22,24,25,65,75,79] and prohibitive environmental characteristics [22,33,54,68] each occurred 8 times (8.16%). Lack of leadership training [22,24,28,29,31] and business aspects interfering with care [80] occurred 7 times respectively (7.14%). High coworker conflicts occurred 6 times (6.12%) [34,35,46,47,78]. Negative perceptions about coaching [31,35,39,72] and Stress [27,30,67] both occurred 5 times (5.10%). Lack of access to management [22,35,41,47] occurred 4 times (4.08%). Poor compensation and benefits [38,39] occurred 3 times (3.06%). Lack of peer support [22,48] and patient morbidity [50,59] occurred 2 times (2.04%). Limited communication opportunities with leadership and team members [22], language barriers [58], non-profit ownership [77], and patient complexity [32] all appeared once (1.02%). For clarification purposes, limited communication opportunities with leadership and team members were identified to decrease job satisfaction [22]. These results provided evidence to meet the two objectives of this study: (1) to provide a summary of facilitators and barriers of associate satisfaction in long-term care facilities, and (2) to provide a summary of the best practices that will benefit long-term care leaders and organizations in the industry. Best practices will be further discussed in the next section.

## 4. Discussion

This study provided a more comprehensive and extensive review in comparison to studies over the previous 5 years than previous studies in long-term care. Because of the extensive nature of this particular review, leaders had the opportunity to prioritize best practices and systems to enhance their own organizations and facilities. Focusing on the top categories of both facilitators and barriers will help long-term care facilities and organizations provide environments that promote both associate and resident satisfaction, while reducing costs associated with unhappy associates and residents.

### 4.1. Most Impactful Themes

Through the systematic review of the 60 selected articles, 162 occurrences of facilitators and 98 occurrences of barriers emerged. The 162 occurrences were grouped into 11 different themes for the facilitators. The eleven “facilitator” themes as demonstrated in Table 2 and represented in Figure 1 included supportive leadership [22,23,24,25,26,27,28,29,30,31,32,33,34,35,36,37,38,39,40,41,42,43,44,45,46,47,48], capable and motivated employees [22,27,30,32,35,45,49,50,51,52,53,54,55,56,57,58,59,60], positive organizational values [22,23,24,29,31,32,33,48,51,53,54,57,61,62,63,64], social support mechanisms, adequate job resources [32,38,45,47,54,55,69,70,71], career/professional development [25,35,37,38,43,48,57,72,73], initial orientation and training [22,38,58,64,68,74], patient centered philosophy [22,28,48,73,75], enjoyment of relationships with patients [25,39,40,48,72,76], non-profit ownership [53,55,63], and organizational systems and processes [22].

**Figure 2:**
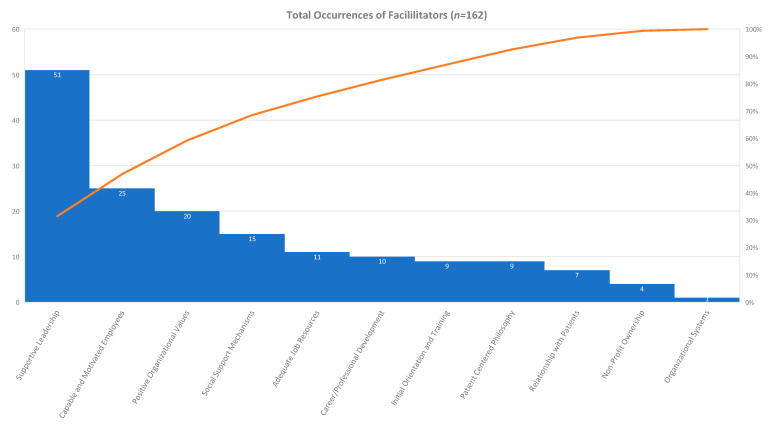
Total occurrences of facilitators in the review with the first four facilitators comprising 68.52% of the occurrences.

As evident in Figure 2, 68.52% of the occurrences grouped into the top four themes of supportive leadership, capable and motivated employees, positive organizational values, and social support mechanisms. One example of supportive leadership stated that employees with high job satisfaction “perceive that their leaders both support them and recognize their job contributions” [47]. One example of capable and motivated employees stated that greater job satisfaction was significantly associated with increased professional efficacy [54]. An example of positive organizational values was found in Gray [48], which states that strong teams, defined as groups that do a good job, cooperate, have mutual respect for one another, and are positively regarded by others, were a source of associate job satisfaction. Social support was found to have a positive influence on employees, improve their sense of autonomy and meaning, and reduce their stressors leading to burnout [34]. Additional examples of themes in the analyzed articles can be found in Appendix A. Enhancing supportive leadership, hiring capable team members, reinforcing capable and empowered associates, grounding a culture in values, and providing support can serve as the key drivers of job satisfaction towards enhanced organizational performance for leaders in the long-term care industry.

**Figure 3:**
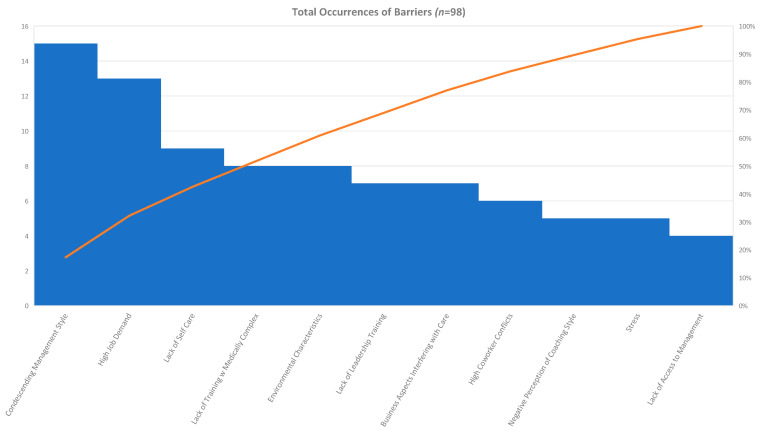
Total occurrences of top barriers in the review with the first six barriers comprising 60.82% of the occurrences.

The 98 occurrences of barriers noted in this study fell into 18 categories, including condescending management style [27,34,35,38,41,42,48,51,66,71,77], high job demands [25,38,40,43,46,65,67,71,72,77,78], lack of self-care by employees [30,32,33,46,47,54,59], lack of training with medically complex patients [22,24,25,65,75,79], lack of leadership training [22,24,27,28,29,31], prohibitive environmental characteristics [22,32,54,68,78], high coworker conflicts [34,35,46,47,76], perception of business aspects interfering with care [24,80,81], negative perceptions about leadership style [31,35,39,72], lack of access to management [22,35,41,47], stress [27,30,42,67], lack of peer support [22,48], patient morbidity [50,59], poor compensation and benefits [38,39,78], limited communication opportunities with leadership and team members [22], language barriers [59], non-profit ownership [77], and patient complexity [32]. The top themes are displayed in Table 2 and Figure 3.

The top four “barrier” themes (condescending management style, high job demands, lack of self-care, and lack of training with the medically complex patients) represented 45.92% of the total occurrences for barriers. An example of condescending management style included the withholding of information by supervisors or coworkers that leads to negative work performance [46]. An example of high job demands included heavy workload, which leads to higher stress levels and lower levels of job satisfaction [49]. Lack of self-care can affect job satisfaction through issues such as brief lunch breaks, low access to healthy foods at work and “working to the point of exhaustion” that can cause employees to be tired and too exhausted to exercise or cook a heathy meal after work [30]. Lack of training with medically complex patients can include young nurses with “less knowledge and experience with elderly care”, which can lead to unrealistic expectations, leading to burnout and decreased engagement [25]. Additional examples of themes in the analyzed articles can be found in Appendix A. If the themes of lack of leadership training and prohibitive environmental characteristics were added to the top four themes, the top six “barrier” themes accounted for 61.22% of the occurrences. Administrators have the opportunity to further demonstrate their commitment to supportive leadership, empowerment of team members, positive organizational values, and social support mechanisms through managing the barriers in long-term care facilities.

### 4.2. Most Impactful Categories of Themes

**Figure 4:**
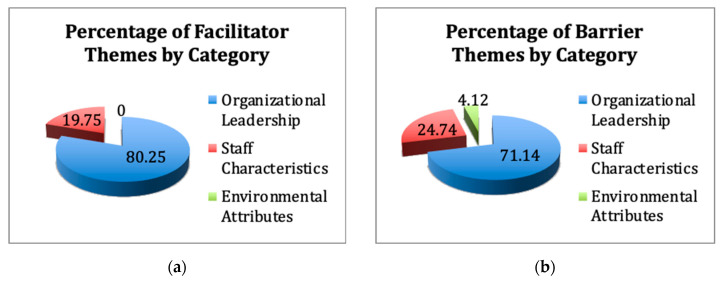
(**a**,**b**) Percentage of facilitators and barriers by theme category.

The researchers noted how the themes grouped into three broad categories pertaining either to the organizational leadership, systems, or processes; to the attributes of team members, associates, or employees; and lastly to the environmental characteristics of long-term care or the nature of the work; depicted in Figure 4a,b.

#### 4.2.1. Organizational Leadership

Nine of the 12 “facilitator” themes (80.25% of the occurrences in the literature) and 10 of the 18 “barrier” themes (71.14% of the occurrences in the literature) represented organizational leadership, systems, or processes. Administrators and organizational leaders have the most control over these organizational themes’ attributes. Within the category of which administrators have the most influence, supportive leadership accounted for 31.48% of the occurrences of facilitators. The findings of this study relative to the long-term care environment [22,23,24,25,26,27,28,29,30,31,32,33,34,35,36,37,38,39,40,41,42,43,44,45,46,47,48,49,50,51,52,53,54,55,56,57,58,59,60,61,62,63,64,65,66,67,68,69,70,71,72,73,74,75,76,77,78,79] reinforced the importance of leaders providing positive and empowering guidance, setting up systems to support team members, providing robust orientation and training on the specific needs of the medically complex population, and reinforcing the positive aspects both in the staff and in the environment, consistent with literature and findings in hospital and healthcare systems [7,8,9,10,11,12,13,14,15]. Supportive leadership was the most cited, influential, and positive facilitator [22,23,24,25,26,27,28,29,30,31,32,33,34,35,36,37,38,39,40,41,42,43,44,45,46,47,48] in long-term care settings, along with setting up systems, processes, and tools to better support associates [22,23,25,29,30,34,41,45,47,65,66,67,68], setting a positive culture [22,23,24,29,31,32,33,48,51,53,54,57,60,61,62,63,64], and inspiring employees [22,26,30,32,35,45,49,50,51,52,53,54,55,56,57,58,59,60].

The most impactful barrier of high job demands [25,38,40,43,46,65,67,71,72,77,78], including staffing [38,46,67], workload [38,78], lack of resources [40,43,46], and the impact of regulatory requirements [72], can be alleviated by a leadership style that integrates listening, frequent communication, trust, and transparency, identified in related hospital and healthcare system literature [7,8,9,10,11,12,13,14,15]. In addition, a process improvement technique, such as the Kaizan approach [82], has value in engaging employees and increasing autonomy on job structure and solutions.

#### 4.2.2. Staff Characteristics

The second-most influential category of themes relates to staff/employee/team member/associate/workforce attributes. Two “facilitator” themes (19.75% of the occurrences in the literature) and five (27.54% of the occurrences in the literature) “barrier” themes relate to staff characteristics. Capability and motivation of employees and team members enjoying their professional relationships with residents or patients represent the inherent attributes that influence associates’ satisfaction [22,25,26,30,32,35,39,40,45,48,49,50,51,52,53,54,55,56,57,58,59,60,72,76]. Consistent with previous work [7,8,9,10,11,12,13,14,15] in healthcare, a long-term care administrator’s best way of supporting associates’ capabilities and enhancing motivation is through adopting a supportive and positive leadership style [7,8,9,10,11,12,13,14,15], the most predominant organizational characteristic influencing satisfaction as per the results in this study. A lack of self-care [30,32,33,46,47,54,59], negative perceptions about a coaching style used by leadership [31,35,39,72], and stress [27,30,43,67] represented intrinsic staff characteristics that have the potential to respond to an administrator who provides support systems such as wellness programs, inviting breakrooms with nutritious snacks, and an open communication style, inviting feedback [7,8,9,10,11,12,13,14,15]. Open, transparent, and frequent communication promoted as the best practice in hospital and healthcare system literature [7,8,9,10,11,12,13,14,15] would serve effectively in alleviating barriers in long-term care as identified in this study [22,23,24,25,26,27,28,29,30,31,32,33,34,35,36,37,38,39,40,41,42,43,44,45,46,47,48,49,50,51,52,53,54,55,56,57,58,59,60,61,62,63,64,65,66,67,68,69,70,71,72,73,74,75,76,77,78,79,80,81].

#### 4.2.3. Environmental Attributes

The least influential of the broad categories of themes was environmental attributes, aspects of providing care in long-term care facilities with the clinical complexity of the population. None of the “facilitator” themes were classified as environmental attributes and 4 of the 18 (24.49%) “barrier” themes were classified as environmental. Patient morbidity [50,59], limited communication opportunities with leadership and team members [22], and patient complexity [32] were included in a few of the articles reviewed as posing barriers to associate satisfaction. Considering how an administrator best manages environmental attributes relative to overcoming these barriers, one could posit again that creating a positive supportive leadership style, establishing positive organizational values, and providing social support mechanisms will most influence how associates react to the environment. Interventions such as including training in techniques to cope with patient morbidity in order to better communicate and gain competence with managing patient complexity, represent examples of how an administrator can best provide support mechanisms for employees [7,8,9,10,11,12,13,14,15].

### 4.3. Implications for Future Success in Long-Term Care and in Times of Crisis and Rapid Change

The secondary purpose of the current study was to determine a best-practice checklist for establishing a long-term care setting, which promotes associate engagement and in turn creates a care environment focused on quality care. The study’s findings suggest the best practices that could facilitate such an environment. Through the findings of this study, leaders in long-term settings are guided to engage in establishing supportive leadership. Supportive leadership, the most frequently cited facilitator in the study, results from the career and professional leadership development of administrators, leaders, and staff, the provision of adequate job resources, orientation, and ongoing training, specifically in terms of the medically complex. All the components that contribute to establishing a supportive leadership style were identified as facilitators.

In addition, the second-most cited facilitator of associate engagement is the presence of a capable and motivated healthcare workforce in long-term care. The secret of empowering and motivating associates comes through such facilitators as establishing social support mechanisms (the third most frequent facilitator theme), rewarding patient-centered care and philosophy, and celebrating associates’ enjoyment of their professional relationships with residents, patients, and clients, all of which were identified in this study as best practices.

Positive organizational values were identified as the third contributing facilitator, which occurs through the establishment of organizational systems and processes and a patient-centered philosophy. Organizational leaders have the responsibility and the opportunity to include all four key facilitators by tapping into the contributions of other facilitators or building blocks for success in quality, resident-centered care.

The reader is encouraged to view from the study, results of what not to do to promote associate satisfaction and perception of quality care through the list of 19 barriers or detractors of care. Many of the barrier themes that emerged could be considered as symptoms of a non-supportive or “condescending” management style, the opposite spectrum of a positive, supportive leadership style.

### 4.4. Limitations and Future Research

The present study provided an extensive systematic review for the facilitators and barriers of the promotion of associate satisfaction of recent literature in long-term care facilities. Although the study met the objectives of identifying useful information relative to facilitators to further promote engagement and satisfaction and suggests the best practices for administrators, limitations existed in the present study. Our study did not categorize or quantify the types of long-term settings with each article review. This was beyond the scope of our study. From a counter perspective, not categorizing the settings lends itself to the generalizability of the results in a variety of long-term settings, including nursing homes, assisted living, community settings providing day services, and other service companies. In addition, our study did not collapse all facilitator and barrier themes, which could also be considered a limitation. Some of the themes could have been considered as sub-components of a broader theme. The reviewers set out to identify as many facilitators and barriers so as to lead to as many insights on best practices and systems to promote associate satisfaction, ultimately leading to resident and family satisfaction.

The current study was qualitative in methodology and although the articles themselves and the authors considered relationships, an opportunity exists to further quantify the relationships between facilitators and barriers. One would speculate that administrators and leaders using positive supportive leadership, creating a culture based on positive organizational values, empowering associates, and providing support mechanisms for employees will decrease the likelihood and influence of detractors or barriers such as condescending management styles, high job demands, and lack of self-empowerment. With that said, the current study did not quantify the relationship of the themes or study the correlation or regression of the themes. Thus, the results of this study provide opportunities for such studies in the future, relating to the strength of the relationship between organizational leadership characteristics and associates’ capabilities and motivation.

A related limitation of the current study is that the authors did not have access to specific associate satisfaction data to make further discoveries on the significance of relationships of various themes within the data. Herein lies a future opportunity for additional studies.

## 5. Conclusions

This study resulted in outlining key components to set in place for the promotion of associate satisfaction. The single most important facilitator of associate satisfaction was establishing supportive leadership, followed by empowering and developing employees, and establishing positive organizational values and support mechanisms. From the opposite perspective, administrators and managers using a condescending management style, setting high job demands, not supporting work-life balance or employees’ self-care, and not providing adequate training to staff on how best to care for the medically complex clients promotes dissatisfaction in their associates. Administrators and leaders have the opportunity to utilize and grow the best practices in order to engage their associates, ultimately increasing the perception of the quality of care for their clients and residents. All of the considerations in this study have been become even more critical in the current situation with fighting the Covid-19 pandemic.

## Figures and Tables

**Table 1:** Summary and frequency of facilitators identified as impacting on job satisfaction.

Facilitators	Occurrences by Article Reference Number	Total Occurrences (*n* = 162)	Percent of Occurrences
Supportive Leadership	[22,23,24,25,26,27,28,29,30,31,32,33,34,35,36,37,38,39,40,41,42,43,44,45,46,47,48]	51	31.48%
Capable and Motivated Employees	[22,26,30,32,35,45,49,50,51,52,53,54,55,56,57,58,59,60]	25	15.43%
Positive Organizational Values	[22,23,24,29,31,32,33,48,51,53,54,57,60,61,62,63,64]	20	12.35%
Social Support Mechanisms	[22,23,25,29,30,34,41,45,47,65,66,67,68]	15	9.26%
Adequate Job Resources	[32,38,45,47,54,55,69,70,71]	11	6.79%
Career/Professional Development	[25,35,37,38,43,48,57,72,73]	10	6.17%
Initial Orientation and Training	[22,38,58,64,68,74]	9	5.56%
Patient-Centered Philosophy	[22,28,48,73,75]	9	5.56%
Enjoyment of Relationships with Patients	[25,39,40,48,72,76]	7	4.32%
Non-Profit Ownership	[53,55,63]	4	2.47%
Organizational Systems and Processes	[22]	1	0.62%

**Table 2:** Summary and frequency of barriers identified as impacting job satisfaction.

Barriers	Occurrences by Article Reference Number	Total Occurrences (*n* = 98)	Percent of Occurrences
Condescending Management Style	[27,34,35,38,41,42,46,51,66,71,77]	15	15.31%
High Job Demands	[25,38,40,43,46,65,67,71,72,77,78]	13	13.27%
Lack of Self-Care	[30,32,33,46,47,54,59]	9	9.18%
Lack of Training with Medically Complex Patients	[22,24,25,65,75,79]	8	8.16%
Prohibitive Environmental Characteristics	[22,32,54,68,78]	8	8.16%
Lack of Leadership Training	[22,24,27,28,29,31]	7	7.14%
Business Aspects Interfering with Care	[24,80,81]	7	7.14%
High Coworker Conflicts	[34,35,46,47,76]	6	6.12%
Negative Perceptions about Coaching Style of Leadership	[31,35,39,72]	5	5.10%
Stress	[27,30,43,67]	5	5.10%
Lack of Access to Management	[22,35,41,47]	4	4.08%
Poor Compensation and Benefits	[38,39,78]	3	3.06%
Lack of Peer Support	[22,48]	2	2.04%
Patient Morbidity	[50,59]	2	2.04%
Limited Communication Opportunities	[22]	1	1.02%
Language Barriers	[59]	1	1.02%
Non-Profit Ownership	[77]	1	1.02%
Patient Complexity	[32]	1	1.02%

## Appendix A

**Table A1:** Summary of articles.

Author Last Name/Year	Objective	Sample/Settings	Study Design and Comparison/Analytical Tool	Key Findings	F/B ^1^	Theme
Aloisio/2019 [22]	To identify individual and organizational predictors of job satisfaction of managers in long-term care (LTC) facilities.	168 managers from 76 LTC homes in three Canadian provinces.	Michigan Organizational Assessment Questionnaire Job Satisfaction Subscale was used to measure job satisfaction. Represented secondary analysis of data from Phase 2 of the Translating Research in Elder Care Programme.	This study identified one individual job satisfaction predictor, high efficacy, and three organizational job satisfaction predictors, social capital, leadership, and adequate orientation.	F	Positive Organizational Values
Perreira/2019 [76]	Explore similarities and differences in the work psychology of health service workers employed in LTC and home and community care settings.	276 LTC employees and 184 home health service employees.	A survey was used to collect data. Path analyses and descriptive statistics were conducted.	This study found that a positive work environment promotes job satisfaction.	F	Enjoyment of Relationships with Patients
				A low perception of support and tension between facilities and healthcare service workers were found to be barriers to job satisfaction.	B	High Coworker Conflicts
Malagon-Aguilera/2019 [23]	To analyze the sense of coherence among RNs ^2^ and its relationship with health and work engagement.	109 Nurses working in an LTC setting.	In a cross-sectional study, 109 registered nurses working in an LTC setting responded to a self-administered questionnaire. Multiple linear regression models were used to analyze social support, work-related family conflicts, sense of coherence, self-reported health status, and work engagement variables.	This study found that implementing a program to implement a sense of coherence (SOC) among nurses can contribute to work engagement. A high SOC was associated with adequate socioeconomic level and social support system. Managers providing social support and assigning meaningful tasks contributed to job satisfaction.	F	Positive Organizational Values, Social Support Mechanisms, and Supportive Leadership and Management
Escrig-Pinol/2019 [24]	To gain a more refined comprehension of the different dimensions of the charge nurse role as a central figure in the LTCFs ^3^ analyzed.	10 RN charge nurses from five LTCFs in Ontario, Canada.	Data were collected via semi-structured interviews. A combination of conventional and direct qualitative content analyses was used.	This study found the charge nurses supervisory skills to directly impact team dynamics and relationships. Professional coaching for charge nurses in LTCFs was found to enhance work–life balance. Recognition for responsibilities increases LTC charge nurse job satisfaction.	F	Supportive Leadership and Management and Positive Organizational Values
				Undefined charge nurse roles and lack of training were barriers to job satisfaction.	B	Lack of Access to Management, Perception of Focus on Business Aspects Interfering with Care, Lack of Leadership Training, and Lack of Training with Medically complex patients
Rajamohan/2019 [75]	To understand the relationship between staff and job satisfaction, stress, turnover, and staff outcomes in PCC NH ^4^ settings.	Electronic research databases between 2000 and 2015.	Review electronic research databases published between 2000 and 2015. Cohen-Mansfield’s comprehensive occupational stress model was used to analyze the relationship between job satisfaction, stress, turnover, and staff outcomes in PCC NH settings.	This study identified providing patient centered care and integrating the patient centered care philosophy into the mission and vision of the organization to facilitate job satisfaction.	F	Patient-Centered Philosophy
				Failure to understand patient-centered care philosophy and failure of leadership to support employees in daily activities were barriers to job satisfaction.	B	Lack of Training with Medically Complex Patients, and Perception of Focus on Business Aspects Interfering with Care
Jirkovská/2019 [81]	To verify the Effort–Reward Imbalance (ERI) model, which serves as a concept to map workplace stress on professional caregivers.	265 Czech professionals in 12 facilities providing health and social care services for the elderly.	The verification of the ERI model along with well-being was conducted on the sample.	This article suggests that a barrier of job satisfaction is the challenge of transforming from a traditional care culture to a patient-centered care culture.	B	Business Aspects Interfering with Care
Kim/2019 [34]	To examine the efforts of social support, job autonomy, and job satisfaction on burnout among LTC workers	170 workers across 23 agencies which administrate LTCF in Hawaii.	A convenience sampling method was used to select participants from 23 agencies which administrate LTCF. The Maslach Burnout Inventory was used to measure burnout level. Job satisfaction was measured using the current job satisfaction scale by Kobiyama. Social support was measured using a tool by Poulin and Walter. Descriptive statistics and bivariate correlations were used to describe the sample and to evaluate possible correlations. A multiple regression analysis was performed to study the effects of major independent variables on burnout controlling for sociodemographic variables.	This study found that a supportive work environment, social support, and a sense of belonging and support from management was key.	F	Social Support Mechanisms, Social Support Mechanisms, and Supportive Leadership and Management
				Lower social support, lower job autonomy, lower administrative support, and a sense of isolation were barriers to job satisfaction.	B	High Coworker Conflicts and Condescending Management Style
Kusmaul/2019 [60]	Identify factors nursing homes can adjust to improve culture for CNAs ^5^ and residents.	106 CNAs employed in 3 LTCF.	A secondary analysis of data gathered from a multi-component paper survey of CNAs employed in LTC. Used results from the Nursing Home survey on Patient Safety Culture and primary shift, type of unit, and years as a CNA to identify modifiable characteristics that would explain variability in the patient safety culture perceptions.	CNAs perception of organizational culture change contributed to job satisfaction.	F	Capable Motivated Employees
Rao/2019 [37]	Identify the conditions in which the impact of hospital nurse staffing, nurse education, and work environment are associated with patient outcomes.	1,262,120 general, orthopedic, and vascular surgery patients, and a random sample of 39,038 hospital staff nurses across 665 hospitals in four large states.	30-day inpatient mortality and failure-to-rescue were the measured outcomes.	Higher professional support and support from colleagues were found to be facilitators to job satisfaction.	F	Career/Professional Development and Supportive Leadership and Management
Desveaux/2019 [52]	To qualitatively determine whether, how, and why an academic detailing intervention could improve evidence uptake, and to identify changes that occurred to advise outcomes for quantitative evaluation.	11 clinical and administrative leaders, 10 physicians, 6 direct care providers, and 2 pharmacists across 13 nursing homes.	A qualitative evaluation of 29 interviews with nursing home staff, which were analyzed using the framework method.	A flexible approach, active knowledge dissemination, and in-person engagement are key components required to drive change.	F	Capable and Motivated Employees
Hartmann/2018 [73]	To improve resident engagement.	Six Veterans Health Administration nursing homes.	A mixed-methods study. The intervention was implemented by using evidence-based tactics for implementing quality improvement and combining CLC-based staff facilitation with researcher-led facilitation. Intervention success was assessed via structured observations and resident and staff surveys collected pre- and post-intervention.	The study found that staff job satisfaction was related to positive meaningful engagements with residents and professional development opportunities.	F	Patient-Centered Philosophy and Career/Professional Development
Huang/2018 [53]	To examine how the presence of owner-managers relates to the workforce outcomes of retentions and wages in Nursing Homes.	For-profit nursing homes in Ohio.	A multiple regression analysis compared workforce outcomes in facilities operated by owner-managers and salaried managers.	This study found that administrative tenure, managerial ownership, supportive environments, managerial involvement in day-to-day operations, non-profit ownership, and feeling empowered leads to greater job satisfaction.	F	Non-Profit Ownership, Capable and Motivated Employees, Non-profit Ownership, and Positive Organizational Values
Whitney/2018 [26]	To further the understanding of the work psychology of health support workers in LTC setting	Ontario, Canada Health service workers working in LTC	A path analysis of data collected from a survey given to the participants.	Health service workers’ (HSW) work outcomes are directly related to work attitudes, work engagement, and organizational commitment. These in turn are related to how HSWs perception of supervisory support.	F	Supportive Leadership
Wagner/2018 [51]	Identify the care provider’s perceptions of spirit at work.	Licensed and unlicensed care providers working in continuing care environments.	Descriptive, mixed-methods study; 18 Likert scale survey questions were further informed by two open-ended questions.	This study suggests that employees with a stronger passion and sense of trust had an increased job satisfaction.	F	Capable and Motivated Employees and Positive Organizational Values
				Management’s inability to resolve problems was a barrier to job satisfaction.	B	Condescending Management Style
Keisu/2018 [36]	Estimate the potential associations between employee-perceived transformational leadership style of their managers, and employees’ ratings of effort and reward within geriatric care work.	RNs, occupational therapists, physiotherapists, and assistant nurses across 9 elderly care facilities in Sweden.	Questionnaires were distributed to participants during an on-site visit. The focus was on balance at work, rather than imbalance.	This study suggests that motivating, inspirational, coaching, and intellectually stimulating managers are facilitators of job satisfaction.	F	Supportive Leadership and Management
Bernstein/2018 [35]	To identify ways to minimize turnover by keeping staff happy in LTC.	N/A	N/A	This study found employee enthusiasm, transparent workplaces, motivating managers, recognition from management, and sense of ownership to increase job satisfaction.	F	Capable and Motivated Employees, Supportive Leadership and Management and Career/Professional Development
				Barriers to job satisfaction included staff feeling like they are not treated fairly, standards not being met, watching the clock, and tension among employees.	B	Condescending Management Style, Negative Perceptions about Coaching Style of Leadership and High Coworker Conflicts
Saito/2018 [25]	Examine work engagement and burnout among nurses in long-term care hospitals in Japan and their relation to nurses’ and organizational values, and congruence of the values.	Nurses in LTC hospitals.	A cross-sectional survey of nurses in long-term care hospital and regression analyses was conducted.	This study found that selfless managers, nurses’ perception of value in their work, nurses’ perception of support, and professional development opportunities contributed to job satisfaction.	F	Supportive Leadership and Management, Enjoyment of Relationships with Patients, Social Support Mechanisms and Career/Professional Development
				Nurses lacking knowledge and preparation, physical burdens, and managers not offering the appropriate support are contributors to burnout and lower engagement.	B	High Job Demands
Backman/2018 [29]	To explore the relationship between the leadership of managers, job strain, and social support as perceived by direct care staff in nursing homes.	3605 nursing home staff members.	A cross-sectional design was used. Participants completed surveys including questions concerning staff characteristics, reliable measures of nursing home managers’ leadership, job strain and social support. Statistical analyses of correlations and regression analysis were also conducted.	The positive leadership of nursing home managers was associated with a lower level of job strain and a higher level of social support among direct care staff.	F	Supportive Leadership and Management, Social Support Mechanisms and Positive Organizational Values
				A barrier to job satisfaction was managers not taking responsibility in supporting and developing strategies to create a healthy working environment.	B	Lack of Leadership Training
Cummings/2018 [31]	To test a model of nursing home staff perceptions of the work context, managers’ use of coaching conversations, and use of research.	33 nursing home managers across seven Canadian nursing homes.	Participants attended a 2-day coaching development workshop. Data were collected via survey. A structural equation modeling was used to test the theoretical model of contextual characteristics of staff use of research, job satisfaction, and burnout as outcome variables and causal variables, managers’ characteristics, and coaching behaviors as mediating variables.	This study found that emotionally intelligent leadership practices, and management’s desire to support staff, were related to job satisfaction.	F	Supportive Leadership and Management and Positive Organizational Values
				Coaching conversations and receiving feedback were found to be associated with decreased job satisfaction.	B	Negative Perceptions about Coaching Style of Leadership
Backhaus/2018 [69]	To understand how nursing homes employ baccalaureate-educated RNs and how the contributions of the RNs to staff and residents in their organizations are viewed.	A combination of board, management, and staff in six nursing home organizations in the Netherlands	A qualitative study was conducted, consisting of 26 individual and group interviews at the board, management, and staff-level in six nursing home organizations in the Netherlands.	Organizations hired baccalaureate-educated RNs to serve as an informal leader for direct care teams. Baccalaureate-educated RN’s roles were unable to be expressed by organizations who did not employ them. Difficulties the RNs experienced during role implementation depended on role clarity, the term used to identify them, support received, transparency from direct care teams, and the RN’s own behavior. The perceived contribution of baccalaureate-educated RNs differed between organizations.	F	Adequate Job Resources
Bethell/2018 [44]	Examine the relationship between supervisory support and intent to turn over amid personal support workers in LTC homes and identifying whether the association is influenced by job satisfaction and the possible effect of happiness.	5645 personal support workers in 398 LTC homes in Ontario Canada	Cross-sectional survey data from the sample was obtained and analyzed through a series of multilevel regression models.	Supervisory support on intent to turn over is mediated by job satisfaction.	F	Supportive Leadership and Management
Yepes-Baldó/2018 [62]	Examine the effects of job crafting activities of elder care and nursing home employees on their perceived well-being and quality of care.	530 elderly care and nursing home employees in Spain and Sweden	Questionnaires on the Job Crafting, the General Health, and the Quality of Care were administered to participants. Correlations and hierarchical regression analyses were also performed.	A positive relationship was found between job crafting and well-being.	F	Positive Organizational Values
Myers/2018 [49]	To understand how LNFAs ^6^ thrive and keep up role performance in stressful SNF environments.	18 LNFAs with an average of 24 years of experience.	A quantitative and qualitative analysis of interviews with participants were conducted by the research team.	This study suggests that high quality skills and safety training, exceptional health benefits, and professional development opportunities increase job satisfaction among CNAs.	F	Initial Orientation and Training, Adequate Job Resources, and Career/Professional Development
				Barriers to job satisfaction included lack of recognition for accomplishments, heavy workloads, and lower pay.	B	Condescending Management Style, High Job Demands and Poor Compensation and Benefits
Berridge/2018 [55]	Examine whether staff empowerment practices common to nursing home culture change are associated with CNA retention	2034 nursing home administrators	Ordered logistic regression was used and data from 2034 nursing home administrators from a national nursing home survey was analyzed.	A high staff empowerment practice score and greater CNA empowerment opportunities were positively associated with greater retention.	F	Capable and Motivated Employees, Adequate Resources to Do the Job and Non-Profit Ownership
Matthews/2017 [27]	To examine the quality of manager–subordinate relationships using Leader-Member Exchange Theory (LMX) as a predictor to turnover among low-wage earners in the LTC environment.	Participants were from a large vertically integrated southeastern LTC organization.	A cross-sectional method was used to gather survey data over two periods. At time one, LMX, demographic information and job satisfaction was measured. At time two, turnover was measured.	This study suggests teamwork, support systems, versatility of foreigners, adequate work time, and working for larger organizations are associated with a higher job satisfaction.	F	Capable and Motivated Employees, Social Support Mechanisms and Adequate Job Resources
				Poor resource management and an unclear base pay amount are barriers to job satisfaction.	B	High Job Demand sand Poor Benefits and Compensation
Doran/2017 [30]	Examine the intrapersonal, interpersonal, and organizational factors that predicted job satisfaction for LTC employees	Long-term care employees.	A forced linear regression model was used, while controlling for age and job title, higher physical activity levels, fewer symptoms of depression, stress, and/or anxiety, less back pain, stronger social support, and reports of low work demands were assessed.	The study found that the main factor associated with job satisfaction was mood, followed by interventions to increase employees’ coping mechanisms and receiving affirmation from management.	F	Capable and Motivated Employees, Social Support Mechanisms and Supportive Leadership and Management
				Barriers to job satisfaction included short lunch breaks and minimal access to nutritious meals.	B	Lack of Self-Care
Pung/2017 [68]	To examine elements of job satisfaction, their demands of immigration score, explore any relationship between job satisfaction and demands of immigration, and determine the predictors of job satisfaction among international nursing staff working in LTC.	International nursing staff group, including those who are non-Singaporean, worked a minimum of one year, and nursing staff who provided direct patient care.	A cross-sectional design was used, and participants were chosen using a convenience-sampling technique.	This study found a positive workplace climate, comprehensive orientation programs, and having the proper resources to succeed were indicators of job satisfaction.	F	Initial Orientation and Training, Social Support Mechanisms, and Adequate Job Resources
				A barrier of job satisfaction is the lack of understanding on the migration demands of nurses and job satisfaction.	B	Prohibitive Environmental Characteristics
Harding/2017 [43]	To determine why staff in care settings are unhappy and understand the benefits of a happier staff.	Long-term care settings and employees in the United Kingdom.	Observations from field experts were included in the study.	This author/expert found that staff development opportunities, supportive managers, and recognition from management increased job satisfaction.	F	Career/Professional Development and Supportive Leadership and Management
				A barrier to job satisfaction was the feeling of the inability to complete tasks in each time frame.	B	High Job Demands and Stress
Boscart/2017 [74]	Changing the impact of nursing assistants’ education in seniors’ care by implementing the Living Classroom (LC) approach.	A Canadian college and nursing home group.	A collaborative approach to integrated learning was conducted where nursing assistant students, college faculty, NH teams, residents, and families engage learning together. This approach placed the student in the nursing home where knowledge, team dynamics, behaviors, relationships, and inter-professional practices are modeled.	Nursing assistant students were highly satisfied with the LC and intention to seek employment in nursing homes have increased. Nursing home teams, residents, and families exhibited positive attitudes towards educating students via LC.	F	Initial Orientation and Training
Chamberlain/2017 [54]	Examine organizational context, care aide characteristics, and frequency of dementia-related resident responsive behaviors associated with burnout.	1194 care aides from 30 urban nursing homes in Canada.	A mixed-effects regression analysis was used to assess care aide characteristics, dementia-related responsive behaviors, unit and facility characteristics, and organizational context predictors of care aide burnout. Burnout was measured using the Maslach Burnout Inventory form.	Unit culture and environmental resources were predictors of professional efficacy, which was associated with increased care aide job satisfaction.	F	Positive Organizational Values, Capable, and Motivated Employees and Adequate Job Resources
				Predictors of emotional exhaustion included English as a second language, medium facility size, organizational slack-staff, organizational slack-space, personal health, and dementia-related behaviors.	B	Prohibitive Environmental Characteristics, Lack of Self-Care
Elliott/2017 [38]	To examine an expanded demand-control-support model that included justice perceptions to determine its impact on multiple types of psychological and organizational well-being outcomes.	173 aged care nurses.	A self-report survey was used to collect and analyze data using hierarchical multiple regression.	This study found job control, high quality training, exceptional health benefits, and professional development opportunities increase job satisfaction.	F	Adequate Job Resources, Adequate Job Resources, Initial Orientation and Training, Adequate Job Resources and Career/Professional Development
				Barriers to job satisfaction included staffing shortages, lack of respect and recognition, low pay, and heavy workload.	B	High Job Demands, Condescending Management Style, High Job Demands and Poor Compensation and Benefits
Adams/2017 [45]	To analyze staff perceptions of skills required and to identify discrepancies in job satisfaction, motivation, and characteristics of staff working in traditional nursing home environments and patient-centered small-scale environments.	A secondary data analysis was conducted from a previous, larger study testing the effects of small-scale living (Verbeek et al., 2009); 138 staff members were included.	A questionnaire was used to gather data on the job satisfaction, motivation, and job characteristics of nursing staff working in small-scale and traditional care environments. Descriptive statistics were used to analyze data, and multilinear regression analysis was used to test the differences between job satisfaction, motivation, and job characteristics.	In small-scale nursing homes, job satisfaction and job motivation were significantly higher compared to those in traditional nursing homes. Job autonomy and social support were also higher, while job demands were lower in small-scale nursing homes. Social support was the most notable predictor of job motivation and job satisfaction in both types of nursing home. Employee patience and motivation was a factor when determining cases where there was an intention to switch care environments.	F	Adequate Job Resources, Social Support Mechanisms, Capable and Motivated Employees and Supportive Leadership and Management
Tong/2017 [46]	To examine the frequency of mobbing in nursing homes and its relationships with care workers’ health status, job satisfaction, and intention to leave, and to examine the work environment as a contributing factor to mobbing.	162 nursing homes in Switzerland with 20 or more beds, including 5311 care workers.	A cross-sectional, multi-center sub-study of the Swiss Nursing Homes Human Resource Project (SHURP). Generalized estimation equations were used to assess the relationships between mobbing and care workers’ job satisfaction, health status, desire to leave, and association of work environment factors with mobbing.	This study found that supportive leadership and open communication contributed to job satisfaction.	F	Supportive Leadership and Management
				Employees affected by mobbing felt leadership was not supportive, felt isolated, and experienced a higher workload, which lead to job dissatisfaction.	B	High Job Demands, High Coworker Conflicts and Condescending Management Style
Chamberlain/2016 [32]	To determine the organizational and individual variables associated with job satisfaction in care aides.	1224 care aides from 30 LTCF homes in three Western Canadian provinces.	Participants reported job satisfaction and perception of the work environment via survey. A hierarchical, mixed-effects ordered logistic regression was used to demonstrate the odds of care aide job satisfaction for individual, care unit and facility factors.	This study found the following factors in the organizational contexts to be associated with higher care aide job satisfaction: leadership, culture, social capital, organizational slack-staff, organizational slack-space, and organizational slack-time.	F	Capable and Motivated Employees, Positive Organizational Values and Adequate Job Resources
				The barriers to job satisfaction were emotional exhaustion and working in Alzheimer’s units.	B	Patient Complexity, Prohibitive Environmental Characteristics and Lack of Self-Care
Schwendimann/2016 [47]	Describe job satisfaction among care workers and to examine its associations with work environment factors, work stressors, and health issues in Swiss nursing homes.	162 Swiss nursing homes including 4145 care workers.	Care worker-reported job satisfaction was measured with a single item. Explanatory variables were assessed with established scales. Factors related to job satisfaction were examined using Generalized Estimating Equation models.	This study found work environmental factors, clear leadership, adequate resources and staffing, supportive leadership, and teamwork to contribute to job satisfaction.	F	Adequate Job Resources, Supportive Leadership and Management and Social Support Mechanisms
				Job satisfaction decreased when workplace conflict increased, during emotional exhaustion, and when access to managing decreased.	B	High Coworker Conflicts, Lack of Self Care and Lack of Access to Management
Wendsche/2016 [77]	To investigate how two types of care settings and types of ownership of geriatric care services influenced RNs intention to change professions.	304 RNs working in 78 different care units in Germany.	A cross-sectional study was conducted collecting questionnaire data from RNs working in 78 care units.	This study found higher job demands, working in for-profit environments, and lack of job control to decrease job satisfaction.	B	High Job Demands, Non-Profit Ownership and Condensing Management Style
Roen/2016 [28]	To examine the association between patient-centered care and organizational, staff and unit characteristics in nursing homes	175 nursing home staff in Norway	A survey was distributed including measures of patient-centered care and questions concerning staff characteristics and work-related psychosocial elements. Association with patient-centered care was analyzed using multilevel linear regression analyses.	High levels of patient-centered care, a patient-centered care work environment, and supportive leadership were associated with greater job satisfaction.	F	Patient-Centered Philosophy and Supportive Leadership and Management
				A barrier to job satisfaction was lack of managerial leadership.	B	Lack of Leadership Training
Gray/2016 [48]	Identify themes to potentially help CNAs make meaning out of their chosen career; thus, potentially explaining increases in job satisfaction among this group.	CNAs at three LTCFs.	Focus groups were conducted with CNAs in the three LTCFs.	This study found strong teamwork, perception of good work, making positive impacts on patients’ lives, positive relationships with residents, professional development opportunities, and recognition by leadership to result in high job satisfaction.	F	Positive Organizational Values, Patient-Centered Philosophy, Enjoyment of Relationships with Patients, Supportive Leadership and Management and Career/Professional Development
				Lack of teamwork resulted in lower job satisfaction.	B	Lack of Peer Support
der Zijpp/2016 [66]	To describe the interaction between managerial leaders and internal facilitators (clinical leaders acting as facilitators) and how this enabled or hindered the facilitation process of implementing urinary incontinence guideline recommendations in a local context in settings that provide LTC to elderly individuals.	105 managers and 22 internal facilitators across four European countries.	Semi-structured interviews were conducted to collect a realist evaluation process. An interpretive data analysis unpacks interactions between managerial leaders and internal facilitators.	This study suggests that building relationships, encouragement from management, and a sense of being valued promote job satisfaction.	F	Social Support Mechanisms
				A lack of commitment from management was a barrier to job satisfaction.	B	Condescending Management Style
Kim/2016 [64]	Analyze the relationship between organizational structure (centralization, formalization, and span of control) and HR practices (training, horizontal communication, and vertical communication) on DCW’s job satisfaction and turnover intent.	58 LTCFs across five states.	A latent class analysis was used to group 58 LTCF characteristics into three combination sets: “organic”: mechanistic”, and “minimalist”. The relationship of each group on direct care worker’s job satisfaction and turnover intent was tested with multivariate regression.	This study found that high levels of job training, increased communication, and higher retention lead to job satisfaction.	F	Initial Orientation and Training and Positive Organizational Values
				A lack of training and communication can serve as a barrier to job satisfaction.	B	Lack of Training with Medically Complex Patients
Kirkham/2016 [40]	Gain an understanding of participants’ lived experiences related to work environment quality and its link with retention; use the knowledge gained to develop a definition of work environment quality from a nursing faculty perspective; and generate grassroots recommendations that can serve as a motive for organizational change.	LPNs ^7^, BSNs ^8^, and professional nurses with a degree from a university in British Columbia.	A participatory action research method called photovoice was utilized.	This study found a stimulating physical work environment and collaborative leadership to improve job satisfaction.	F	Enjoyment of Relationships with Patients and Supportive Leadership and Management
				A barrier to job satisfaction is an inadequate amount of time to fulfil required duties.	B	High Job Demands
Ginsburg/2016 [56]	Validate three key work attitude measures to better understand health care aids work experiences: work engagement, psychological empowerment, and organizational citizenship behavior.	306 health care aides working in LTC and home and community care.	Data were collected from health care aides working in LTC and home and community care using work engagement, psychological empowerment, and organizational citizenship behavior surveys. Psychometric evaluation consisted of confirmatory factor analysis. Predictive validity and internal consistency reliability were examined.	This study found work engagement, positive work attitudes, and incentive systems to predict job satisfaction.	F	Capable and Motivated Employees
Jenull/2015 [59]	Identify sharp patters of work stress among nurses.	844 nurses.	Latent profile analyses were used to determine distinct patterns of work stress. Sociodemographic variables such as nurses’ living conditions and reactions to workload were considered to predict the participants’ profile membership.	Community connectedness and higher quality of life were positively associated with job satisfaction.	F	Capable and Motivated Employees
				Working in a distressing, inpatient environment, high work demands, language barriers, understaffed, and feeling unable to provide patient centered care were barriers to job satisfaction.	B	Patient Morbidity, Language Barriers and Lack of Self Care
Zwijsen/2015 [58]	Determine the effects of a care program for the challenging behavior of nursing home residents with dementia on burnout, job satisfaction, and job demand of care staff.	17 Dutch dementia special care units.	Burnout, job satisfaction, and job demands were measured before the implementation, in the middle of the program, and after every unit implemented in the care program. The Dutch version of Maslach burnout inventory was used to measure burnout. Job satisfaction and demands were measured using subscales of the Leiden Quality of Work Questionnaire. Effects were determined using mixed model analyses.	Notable positive effects of using the grip on challenging behavior care programs were found on job satisfaction, without an increase placed on job demands.	F	Initial Orientation and Training and Capable and Motivated Employees
Thompson/2015 [80]	To consider the influence of multiple-source care funding issues on nursing-home nurses’ job satisfaction.	13 nurses from seven nursing homes in North-East England.	Hermeneutic phenomenology was used in this study. Participants were interviewed in a sequence of five interviews, and the data were analyzed using a literary analysis method.	This study found that tension between funding and care, the feeling of “selling beds,” multiple-source care funding, and difficulty coping with self-funding residents was a barrier to nurses’ job satisfaction in the LTC setting.	B	Business Aspects Interfering with Care
Binney/2015 [50]	Explore the perceptions of work engagement among RNs working in LTC.	Eight RNs in an LTCF in the United States.	Eight RNs in a LTCF in the United States served as subjects in the study.	Dedicated and committed nurses increase job satisfaction.	F	Capable and Motivated Employees
				High patient morality rates, increased workloads, and stress were barriers to job satisfaction.	B	Patient Morbidity
Tsukamoto/2015 [61]	Investigate the associations between emotional labor, general health, and job satisfaction among long-term care workers.	132 established, private day care centers in Tokyo	Cross-sectional study utilizing a mail survey. The outcome variables included two health-related variables and four job satisfaction variables: physical and psychological health, satisfaction with wages, interpersonal relationships, work environment, and job satisfaction. Utilized multiple regression analyses to identify significant factors. Directors from 36 facilities agreed to participate.	Findings indicated that the emotional labor of long-term care workers has a negative and positive influence on health and workplace satisfaction and suggests that care quality and stable employment among long-term care workers might affect their emotional labor.	F	Positive Organizational Values
Knecht/2015 [39]	Examine the attributes of LTC LPNs’ job satisfaction and dissatisfaction.	4–12 LPNs in six LTCFs in Philadelphia.	A qualitative, 90 min focus group study was conducted at each LTCF. Four to twelve LPNs in each of the six focus groups participated in a focus group session. Herzberg’s motivation/hygiene theory (1959) provided the basis for the framework for the study. The focus group methodology allowed for the utilization of the collective power of individual and group discussion, resulting in ample data. Data analysis began after the first focus group session was complete. Utilization of member checks, expert verification, and maintenance of an audit trail contributed to trustworthiness.	This study found that work recognition and feeling valued by residents’ families increased job satisfaction	F	Supportive Leadership and Management and Relationships with Patients
				Barriers to job satisfaction included poor pay and poor performance not being properly reprimanded by management.	B	Negative Perceptions about Coaching Style of Leadership and Poor Compensation and Benefits
Smikle/2015 [57]	To investigate why LTC employees choose to stay and explore retention strategies for LTC.	39 LTC employees with 10–34-year tenure rating.	Participants participated in a single, individual review. Stories relating to why employees chose to stay were gathered.	Facilitators to job satisfaction in this study included involvement in the organization, supportive and compassionate leadership, sense of connectedness, and professional development opportunities.	F	Capable and Motivated Employees, Positive Organizational Values and Career/Professional Development
Wallin/2015 [65]	Investigate job strain and stress of conscience among nurse assistants working in residential care and explore associations with personal and work-related aspects and health complaints.	225 nursing assistants.	Questionnaires to measure job strain, stress of conscience, personal and work-related aspects of health complaints were completed by 225 nursing assistants. Multiple linear regression analyses were performed to compare high and low levels of job strain and stress of conscience.	Organizational and environmental support were positively associated with job satisfaction.	F	Social Support Mechanisms
				Lack of education, poor leadership, heavy workload, and lack of opportunities to discuss difficult situations were found to be barriers to job satisfaction.	B	Lack of Training with Medically Complex Patients and High Job Demands
Butler/2014 [71]	Explore determinants of longer job tenure for home care aides.	261 home care aides.	Mixed-method study. Home care aides were followed for 18 months and completed two mail surveys and one phone interview.	The study found higher wages to be a predicter of job satisfaction.	F	Adequate Job Resources
				Lack of available hours, poor compensation, poor communication, and difficulty building relationships with employing agency contributed to a decrease in job satisfaction.	B	High Job Demands and Condescending Management Style
Zhang/2014 [33]	Identify the relationships among LTC employees’ working conditions, mental health, and desire to leave.	1589 employees from 18 for-profit nursing homes	This is a quantitative study. Data were collected via self-administered questionnaires from 1589 for-profit nursing homes. The number of beneficial job features was constructed via a working condition index.	This study found that healthy working environments, strong interpersonal relationships, coworker support, and supervisor support contributed to job satisfaction.	F	Positive Organizational Values and Supportive Leadership and Management
				Poor mental health was a barrier to job satisfaction.	B	Lack of Self-Care
Wendsche/2014 [63]	Identify direct and indirect links between geriatric care setting, rest break organization, and registered nurses’ turnover assessed for one year.	80 nursing units within 51 geriatric care services employing 597 RNs in Germany.	Multimethod cross-sectional study was used to assess nursing unites within geriatric care.	This study found collective rest breaks and working in non-profit facilities to contribute to job satisfaction.	F	Positive Organizational Values, Non-Profit Ownership
Willemse/2014 [70]	Highlight the consequences of small-scale care on staff’s perceived job characteristics.	136 Dutch dementia care nursing homes with 1327 residents and 1147 staff.	Multilevel regression analyses were used to study the relationship between two indicators of small-scale care and staff’s job characteristics.	Nurses being assigned less residents/patients had a positive effect on job satisfaction in this study.	F	Adequate Job Resources
McGilton/2014 [72]	Understand factors that influence nurses’ intentions to continue employment at their current job.	41 LTC nurses in seven nursing homes in Ontario, Canada.	Focus groups were conducted at the seven nursing homes through focus groups, and the discussions within the group were transcribed verbatim. Themes for the groups were developed via directed content analysis.	This study found the development of meaningful relationships with residents and professional development opportunities to promote job satisfaction.	F	Relationships with Patients and Career/Professional Development
				Regulations on role flexibility, underfunded systems, and lack of management support were barriers to job satisfaction.	B	High Job Demand sand Negative Perceptions about Coaching Style of Leadership
Meyer/2014 [78]	To follow rural CNAs in the US one year after training to identify retention and turnover in the LTC setting, using CAN’s perceptions of the LTC work experience.	123 CNAs from the United States.	A longitudinal survey design was used to track CNAs completing training for one year.	The first 6 months of employment most impacted retention; 53.7% CNAs were retained after one year; and the CNAs leaving cited pay as being the number one reason for leaving.	B	High Job Demands and Poor Comp and Benefits
				Heavy workloads were found to be a barrier to job satisfaction		Prohibitive Environmental Characteristics
McGilton/2014 [42]	Describe the organizational, unregulated nurse and resident outcomes associated with effective supervisory performance of regulated nurses in LTC homes.	Regulated nurses working in LTC homes.	Six databases were utilized to gather articles between 2000 and 2015. Twenty-four articles were selected, and an integrative interview was performed.	Nurse supervisor performance left employees feeling empowered, increasing job satisfaction.	F	Supportive Leadership and Management
				Poor supervision over nurses was a barrier to job satisfaction.	B	Condescending Management Style
Chu/2014 [41]	Describe the relationship between nursing staff turnover in LTC homes and organizational factors (i.e., leadership practices and behaviors, supervisory support, burnout, job satisfaction and work environment satisfaction).	LTCF administrators.	A stress process model was used. Surveys were distributed to LTC administrators to measure organizational factors and to regulated nurses to measure sources of stress and workplace support; 324 surveys were used in a linear regression analysis to examine factors related to high turnover rates.	This study identified greater support from management, empowering work environments, and employee assistance programs as contributors to job satisfaction.	F	Supportive Leadership and Management and Social Support Mechanisms
				Lack of supervisory support, poor leadership, administration turnover, and poor work environments were barriers to job satisfaction.	B	Condescending Management Style by Supervisor or Senior Leaders and Lack of Access to Management
Kuo/2014 [67]	Explore the mediating effects of job satisfaction on work stress and turnover intention among LTC nurses.	LTC nurses in Taiwan.	The study used a cross-sectional survey and a correlation design. Multistage linear regression was utilized to test the mediation model.	Low stress levels among LTC employees lead to a higher job satisfaction.	F	Social Support Mechanisms
				Barriers to job satisfaction include high stress levels, unfair working hours, and lack of supportive leadership.	B	High Job Demands
Jungyoon/2014 [79]	To examine the relationship between organizational structure and HR practices on job satisfaction and turnover intent.	50 LTC facilities across five states.	A latent class analysis was used to group facility characteristics into three sets of combinations. A multivariate regression was used to test the relationship between groups on job satisfaction and turnover intent.	This study found low levels of job training and communication to be barriers to job satisfaction.	B	Lack of Training with Medically Complex Patients

^1^ F = Facilitator and B = Barrier; ^2^ RN = Registered Nurse; ^3^ LTCF = Long-Term Care Facility; ^4^ PCC NH = Person-Centered Care Nursing Home; ^5^ CNA = Certified Nurse Assistant; ^6^ LNFA = Licensed Nursing Facility Administrator; ^7^ LPN = Licensed Practical Nurse; ^8^ BSN = Nurse with a Bachelor of Science in Nursing.
